# Selection of SSR markers for population studies in *Eucalyptus globulus* seed orchards

**DOI:** 10.1186/1753-6561-5-S7-P59

**Published:** 2011-09-13

**Authors:** Diego Torres-Dini, Zohra Bennadji, Laura Lima-Aliano, Natalia Nikichuk, Fernando Resquin, Gustavo Balmelli

**Affiliations:** 1 Forest Biotechnology Laboratory, INIA Tacaurembo, Uruguay; 2 Fosrestry Breeding Program, INIA Tacuarembo, Uruguay; 3 Uruguay.University of the Republic, Montevideo Uruguay

## Background

*Eucalyptus globulus* is a widely planted species in temperate regions of the world for pulpwood production. Its good characteristics for kraft pulping in addition to a broad adaptability to different site conditions have led this species to be employed in commercial plantations and to be included in breeding programs [1]. Uruguay has approximately 300.000 hectares forested with *E. globulus* being the most cultivated species in the country and representing 45% of the total forested area. The main objectives of many Uruguayan breeding programs for *E. globulus* are the increase of volume per hectare, basic density and pulp yield. The strategies for genetic improvement used in many breeding programs in Uruguay require seed multiplication in seed orchards to obtain genetic gain [2].

In long-term breeding programs, the strict selection of reproductive populations restricts the number of genotypes involved in the final orchard, thus reducing genetic diversity and increasing the risk of depression through inbreeding in the following generations. The use of SSRs (simple sequence repeat) markers as selecting strategies of seed orchards in other species such as *Eucalyptus dunni*, has proved to be an excellent tool to reduce inbreeding [3,4]. The transferability of SSR markers across Eucalyptus species has been widely studied [5-8]. The aim of this work was to select highly polymorphic SSR markers of *E. globulus* to assist breeding programs.

## Methodology

Eighteen *E. globulus* samples were collected from the breeding program of the National Agricultural Research Institute of Uruguay .DNA was extracted with CTAB 2X buffer [9]. Eight SSR markers, previously reported for *E. grandis* and *E. urophylla* were used: EMBRA 8, EMBRA 11, EMBRA 18, EMBRA 32, EMBRA 47, EMBRA 51, EMBRA 58 and EMBRA 155 [6,7] The annealing temperature was specifically adjusted for *E. globulus* using a temperature gradient of 52°C – 56°C. The other reaction parameters did not differ from those previously reported. The results were visualized in polyacrilamyde gels 8% under denaturing conditions at 80 W constant power over 3h 30min and were stained with silver nitrate. The estimation of the molecular weight of the alleles was made by comparison with a ladder. The matrix information was analyzed with the software Identity v 1.0 [10] and the following parameters were calculated: alleles number per loci, expected heterozygosity (He), observed heterozygosity (Ho), identity probability (Pi) and paternity exclusion probability (Pe).

## Results and conclusion

From the eight tested markers, EMBRA 8, EMBRA 18 and EMBRA 13 did not amplify at any of the tested temperatures. EMBRA 11 and EMBRA 47 showed good amplification at 52°C of annealing. The rest of the primers worked optimally in the conditions previously described for *E. grandis* and *E. urophylla*[7]. The five SSRs markers amplified a total of 42 alleles. With a maximum of 14 alleles for EMBRA 11, 9 alleles for the markers EMBRA 47 and EMBRA 58 and 5 alleles for the markers EMBRA 51 and EMBRA 155. The range of heterozygosity expected for all the loci ranged from 0.64 to 0.88. However, the observed heterozygosity showed values of 0.1 to 0.9. With this information the probability of identity (IP) was 17 x 10^-5^ and the paternity exclusion probability was 0.99. The considerable number of SSR markers currently published [6-8] confirm how necessary the availability of these tools is to carry out precise population analyses. The five identified markers turned out to be promising candidates to be used in *E. globulus* seed orchards population studies.
